# Approaches to triage optimization in HPV primary screening: Extended genotyping and p16/Ki‐67 dual‐stained cytology—Retrospective insights from ATHENA

**DOI:** 10.1002/ijc.32669

**Published:** 2019-10-06

**Authors:** Mark H. Stoler, Ed Baker, Sean Boyle, Shagufta Aslam, Ruediger Ridder, Warner K. Huh, Thomas C. Wright

**Affiliations:** ^1^ Department of Pathology University of Virginia Health System Charlottesville VA; ^2^ Roche Molecular Systems Inc. Pleasanton CA; ^3^ Ventana Medical Systems, Inc. (Roche Tissue Diagnostics) Tucson AZ; ^4^ Division of Gynecologic Oncology University of Alabama at Birmingham Birmingham AL; ^5^ Department of Pathology Columbia University New York City NY

**Keywords:** triage, HPV, extended genotyping, p16/Ki‐67 dual‐stained cytology, pap cytology

## Abstract

The objective of our study was to assess the performance of different triage strategies for high‐risk human papillomavirus (hrHPV)‐positive results utilizing either extended genotyping or a p16/Ki‐67 dual‐stained cytology (DS) approach, with or without partial genotyping. A subset of women with hrHPV infections participating in the Addressing the Need for Advanced HPV Diagnostics (ATHENA) study were analyzed to determine the number of cervical intraepithelial neoplasia grade 3 or worse (≥CIN3) cases detected, and the absolute risk for ≥CIN3 of each genotype. A clinical utility table was constructed to compare the impact of different triage strategies. In all, 2,339 women with single‐genotype hrHPV infections were identified. Among these were 171 ≥CIN3 cases. The U.S. Food and Drug Administration (FDA)‐approved algorithm (HPV16/18 positive, or 12‐other hrHPV positive and Pap positive, i.e., ≥ atypical squamous cells of undetermined significance) for primary HPV screening detected 132/171 (77.2%) ≥CIN3 cases and required 964 colposcopies (colposcopies per ≥CIN3 ratio: 7.3). An approach that uses DS instead of cytology in the FDA‐approved algorithm detected 147/171 (86.0%) ≥CIN3 cases, requiring 1,012 colposcopies (ratio: 6.9). Utilizing DS for triage of all hrHPV‐positive women identified 126/171 (73.7%) ≥CIN3 cases, requiring 640 colposcopies (ratio: 5.1). A strategy that detected HPV16/18/31/33/35+ captured 130/171 (76.0%) ≥CIN3 cases, requiring 1,025 colposcopies (ratio: 7.9). Inclusion of additional genotypes resulted in greater disease detection at the expense of higher colposcopy ratios. Substituting cytology with a DS triage approach improved disease detection and the colposcopy detection rate. Further reduction of colposcopy rates can be achieved by using DS without partial genotyping. Extended genotyping strategies can identify a comparable number of cases but requires an increased number of colposcopies.

## Abstract 2

**What's new?**

Among HPV‐positive women, the goal is to maximize the number of CIN3 or greater cases detected while minimizing colposcopies. These authors compared various combinations of genotyping and dual‐stained cytology (DS) as triage strategies. From a pool of 2,339 hrHPV+ women, 171 cases with ≥CIN3 were identified. Only one algorithm they tested boosted sensitivity over the current approach without reducing specificity. This method used DS to triage women who tested positive for 12‐other HPV, and referring HPV16/18+ patients directly to colposcopy. This approach caught 147 of the 171 cases, while reducing the number of colposcopies per case from 7.4 to 6.9.

**Abbreviations**
AIS: adenocarcinoma *in situ*
ASCUS: atypical squamous cells of undetermined significance
ATHENA: Addressing the Need for Advanced HPV Diagnostics
CI: confidence interval
CIN: cervical intraepithelial neoplasia
CIR: cumulative incidence rate
CPR: central pathology review
DS: dual stain (p16/Ki‐67 dual‐stained cytology)
FDA: U.S. Food and Drug Administration
HPV: human papillomavirus
hrHPV: high‐risk human papillomavirus
KPNC: Kaiser Permanente Northern California
NILM: negative for intraepithelial lesion or malignancy

## Introduction

There is an evolving worldwide consensus that the best way to screen for cervical cancer is to include high‐risk human papillomavirus (hrHPV) testing in the initial screening process. Multiple studies have documented the superior sensitivity of clinically validated hrHPV testing over Pap cytology for detecting cervical precancer and cancer.^1^, ^2^, ^3^ This high level of sensitivity leads to maximal reassurance for the great majority of women with screen test negative results. Hence, most countries with established screening programs are moving from cytology‐based screening to a system of primary hrHPV testing.^4^ In the United States, cotesting, that is, performing a human papillomavirus (HPV) test and Pap cytology on all screened women in parallel, has been dominant. However, HPV primary screening has been approved for 5 years, and the most recent United States Preventive Services Task Force screening guidelines prefer primary hrHPV testing every 5 years to cytology every 3 years for screening.^5^, ^6^, ^7^

The high degree of reassurance of a negative HPV test result is balanced by the risk of precancer or cancer in HPV‐positive women. The prevalence of HPV infections in a screening population can depend on the age distribution and sexual activity, but averages ~10% in Western countries.^8^, ^9^, ^10^ In contrast, the prevalence of precancer, defined as cervical intraepithelial neoplasia grade 3 (CIN3) and adenocarcinoma *in situ* (AIS), is on the order of 0.5–1%.^11^, ^12^ As it is neither possible nor clinically desirable to refer all HPV‐positive women to colposcopy, a triage strategy for HPV‐positive women is needed to identify the women who would benefit most from immediate referral to colposcopy. For example, in the Netherlands, a target referral rate of 2% of screened women is considered “ideal,” producing a ratio of 2–4 colposcopies per CIN3 detected.^13^

U.S. guidelines from 2012 apply the concept of equal management for equal risk to drive an algorithmic referral strategy for colposcopy.^5^, ^14^ Those having ~5% risk of ≥CIN3, defined as CIN3, AIS, adenocarcinoma and squamous carcinoma, within 3 years of their positive screening test are referred for colposcopic assessment and potential biopsy, whereas those patients whose risk is below that threshold are referred to repeat examination by cotesting within 12 months.^5^ Thus, in all HPV‐based screening programs, the focus is on balancing the risk of identifying precancer/cancer while optimizing disease yield at colposcopy in the screen‐positive population.

Triage of HPV positives can be accomplished in several ways. In almost all current algorithms, Pap cytology is applied to the HPV‐positive individuals, and any abnormal cytology result leads to colposcopy. Cytology has the advantage that it is already available in all established screening systems, and it is relatively specific for colposcopically identifiable disease, especially if the cytology is nonequivocal (i.e., > atypical squamous cells of undetermined significance [ASCUS]).

However, just as cytology is less sensitive as a screening test, it may suffer in sensitivity as a triage test. Indeed, one of the criticisms of HPV primary screening with cytology triage is that the triage step may hinder the sensitivity of the algorithm. Therefore, in many settings, either repeat cytology after 6 months or partial HPV genotyping has been used to improve identification of patients most at risk for ≥CIN3.^15^

In current U.S. practice, partial genotyping means testing for HPV genotypes 16 and 18, which together account for 60–70% of cervical cancers.^16^ In the Addressing the Need for Advanced HPV Diagnostics (ATHENA) clinical study, women who were HPV16 positive and had a normal Pap cytology at baseline had a cross‐sectional risk of ≥CIN3 two to three times that of the colposcopic referral threshold, and after 3 years this risk was fivefold higher.^1^

Extended genotyping generally refers to an approach that uses individual genotype‐specific information beyond HPV genotypes 16 and 18, that is, it offers insight into the amalgamated 12‐other hrHPV genotypes reported as a pooled hrHPV‐positive result by various U.S. Food and Drug Administration (FDA)‐approved HPV tests. Recent epidemiologic data have shown that the risk of CIN3 for some of those 12‐other hrHPV genotypes, such as HPV31 or 33, is on par with HPV18.^11^, ^17^ Therefore, following the concept of equal management for equal risk, questions have arisen as to how extended genotyping might shift the balance among the two screening groups created by triage of screen positives: those referred for immediate colposcopy and those who can safely be deferred for follow‐up testing after 12 months.

Besides cytology and partial genotyping, a promising triage test shown to offer improvement over cytology is p16/Ki‐67 dual‐stain (DS) testing on a liquid‐based cervical cytology specimen.^18^, ^19^, ^20^ DS testing immunocytochemically detects the abnormal coexpression of p16, a tumor‐suppressor upregulated by hrHPV oncogene activity, and the cell proliferation marker Ki‐67. The detection of simultaneous coexpression of the tumor suppressor protein p16 and the proliferation marker Ki‐67 within the same cell is indicative of cell cycle dysregulation associated with transforming HPV infections and cervical neoplasia.^21^ In ATHENA, and other studies, DS testing demonstrated better performance (improved sensitivity and at least equal specificity) compared to cytology for the cross‐sectional detection of ≥CIN3 in hrHPV‐positive women.^20^, ^22^, ^23^, ^24^, ^25^, ^26^, ^27^, ^28^, ^29^, ^30^, ^31^ However, like Pap cytology, DS testing is not integrated into the first‐line HPV screening test, and both Pap cytology and DS testing require the preparation of a slide for subjective microscopic assessment by highly trained professionals.

Current strategies for triage of hrHPV‐positive screening results include cytology, some form of genotyping, DS testing or combinations thereof. There is also growing interest in the potential utility of testing for alterations of both host and viral methylation patterns for triage.^32^, ^33^ Various publications discussing proposed testing strategies and clinical algorithms utilize the sensitivity for ≥CIN3 cross‐sectionally, the number of tests performed, and the number of colposcopies necessary to find a case of ≥CIN3 as measures of algorithm performance.^1^, ^34^ Furthermore, the choice of HPV testing platform may impact the triage test options. For example, the Hybrid Capture 2 HPV test (Qiagen, Hilden, Germany) does not offer partial or extended genotyping, whereas the Aptima® HPV test (Hologic, Marlborough, MA) requires an additional reflex test from the original specimen to perform partial genotyping for HPV16 and 18/45, and the cobas® HPV Test (Roche Molecular Systems Inc, Pleasanton, CA) and the Onclarity™ HPV test (BD Diagnostics, Franklin Lakes, NJ) both offer some degree of integrated genotyping. This leads to the question that we aim to address here: how does extended genotyping compare with DS testing, with or without partial genotyping, when incorporated into a screening strategy as a triage test for hrHPV‐positive screening results? In this analysis, we utilized data from the ATHENA study, with which ≥CIN3 outcomes can be evaluated in the context of extended genotyping and DS test results.

## Materials and Methods

### Study design

The present study expands upon previous analyses from ATHENA, a large U.S.‐based, multicenter cervical cancer screening study in more than 47,000 women. The study demographics, design and genotype‐specific results have been described in detail previously.^8^, ^12^ The study protocol was approved by institutional review boards at all participating centers. All participants provided informed consent before enrollment. The study was registered with https://ClinicalTrials.gov (NCT00709891).

ATHENA has been well described in other publications^12^, ^34^, ^35^, ^36^ but, briefly, the ATHENA study was designed to assess the performance of the cobas HPV Test on the cobas 4800 platform (Roche Molecular Systems Inc), compared with liquid‐based cytology for cervical cancer screening in a large U.S. population aged 21 years and older. Cervical specimens were obtained for liquid‐based cytology and HPV DNA testing with two first‐generation assays (AMPLICOR® HPV Test and LINEAR ARRAY® HPV Genotyping Test; Roche Molecular Systems Inc) and the second‐generation cobas HPV Test (with integrated type‐specific identification of HPV16 and HPV18).^37^ Women with either a positive HPV test and/or abnormal Pap cytology result were referred for colposcopy. Additionally, a random subset of women with negative results from both tests was referred for colposcopy.

Subsequently, 8,067 archived liquid‐based cytology specimens stored at 2–8°C for up to 5 years from women ≥25 years were utilized for p16/Ki‐67 DS testing using the CINtec® PLUS Cytology test (Roche mtm laboratories, Mannheim, Germany). The CINtec PLUS Cytology test was performed as previously described.^38^ This substudy was done to assess the performance of p16/Ki‐67 DS testing for triaging HPV‐positive women ≥25 years old undergoing primary HPV screening in comparison to liquid‐based cytology.^38^

**Figure 1:**
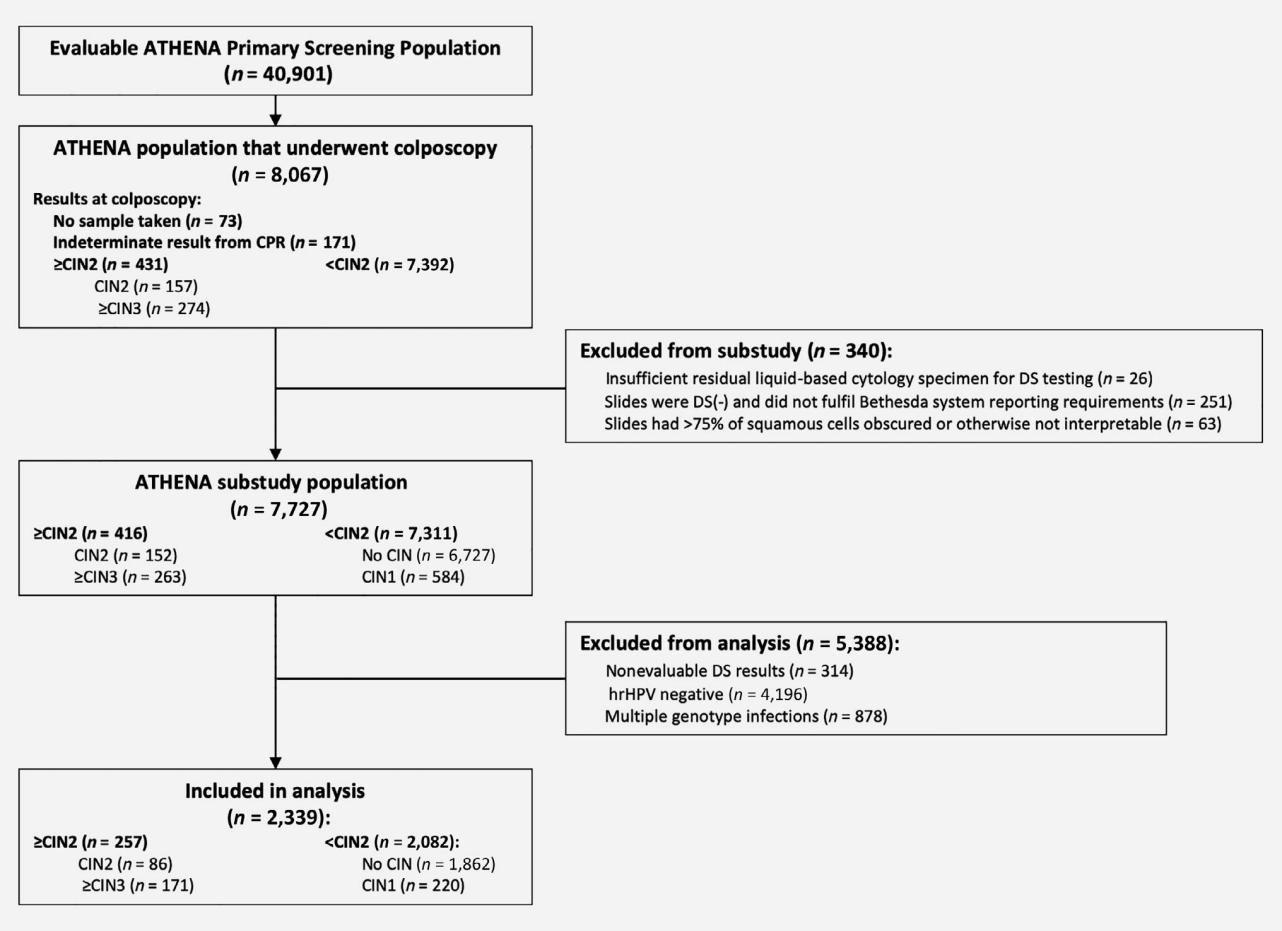
Flowchart demonstrating women ≥25 years old from the previous ATHENA substudy eligible for inclusion in this analysis. The present study evaluated data from the 7,727 patients included in the previous ATHENA substudy.^38^ Women with valid results for cytology, DS testing, HPV testing and genotype testing, with single HPV genotype infections were included in this analysis. Abbreviations: ATHENA, Addressing the Need for Advanced HPV Diagnostics; CIN, cervical intraepithelial neoplasia; CIN1, CIN grade 1; CIN2, CIN grade 2; CIN3, CIN grade 3; CPR, central pathology review; DS, dual‐stain; hrHPV, high‐risk human papillomavirus.

Of the evaluable patients included in the ATHENA substudy population, a further subset of women with single HPV‐positive infections by the LINEAR ARRAY® HPV Genotyping Test, who had valid test results for the cobas® HPV Test, p16/Ki‐67 DS testing, liquid‐based cytology and a valid central pathology review (CPR) result formed the basis of the present analysis (Fig. 1).

### Statistical methods

The cobas® HPV Test result was used to determine if the infection was caused by HPV16 or 18. The LINEAR ARRAY® HPV Genotyping Test result was used to determine which genotype was responsible for a positive result for detection of one of the 12‐other hrHPV genotypes reported as “12‐other hrHPV positive” as a pooled result by the cobas® HPV Test. Results were evaluated to determine the number of single infections by each of the 14 hrHPV genotypes, and to calculate the number of ≥CIN3 cases detected by a positive HPV test result. Absolute risks of ≥CIN3, along with 95% confidence intervals (CIs), were calculated for each single genotype infection at baseline (Table 1).

**Table 1:** Absolute risk for ≥CIN3 in single genotype infection

			Absolute risk	
hrHPV genotype	No. of infections	No. of ≥CIN3 cases	Estimate, %	95% CI
16	431	83	19.3	(15.8–23.3)
18	179	18	10.1	(6.5–15.3)
31	228	17	7.5	(4.7–11.6)
33	62	6	9.7	(4.5–19.6)
35	125	6	4.8	(2.2–10.1)
39	202	9	4.5	(2.4–8.3)
45	151	7	4.6	(2.3–9.3)
51	137	1	0.7	(0.1–4.0)
52	187	17	9.1	(5.8–14.1)
56	118	1	0.9	(0.2–4.6)
58	106	3	2.8	(1.0–8.0)
59	139	0	0.0	
66	157	1	0.6	(0.1–3.5)
68	117	2	1.7	(0.5–6.0)
Total	2,339	171		

The number of single infections by one of any of the 14 hrHPV genotypes is shown along with the number of ≥CIN3 cases detected by a positive HPV test result and the absolute risks of ≥CIN3 for each single genotype infection at baseline. Genotypes 16 and 18 were identified using the cobas HPV Test and the 12‐other HPV genotypes were identified using the LINEAR ARRAY HPV Genotyping Test.

Abbreviations: CI, confidence interval; ≥CIN3, cervical intraepithelial neoplasia ≥ grade 3; hrHPV, high‐risk human papillomavirus; NA, not applicable.

The performance of each of 10 potential cervical cancer triage strategies was compared with the performance of the existing FDA‐approved algorithm (HPV16/18 positive, or 12‐other hrHPV positive and Pap positive, i.e., ≥ASCUS) by using the ratio of sensitivities and 1‐specificities of a potential strategy relative to the existing FDA‐approved algorithm.^39^ SE and SE0 are the sensitivities of a potential strategy and existing strategy, respectively. SP and SP0 represent the specificities of a potential strategy and existing strategy, respectively. Rx = SE/SE0 and Ry = (1‐SP)/(1‐SP0) are the ratios of sensitivities (ratio of true positive rates) and 1‐specificities (ratio of false positive rates), respectively. The difference between Ry and Rx can be considered as a measure of overall effectiveness of a potential strategy relative to the existing FDA‐approved algorithm. For each potential strategy, the difference between Ry and Rx were calculated along with 95% CIs.^40^ CIs not including “0” indicate statistical significance.

Sensitivity and specificity, along with 95% CIs, were plotted for each screening strategy. To compare the impact of various triage strategies on baseline disease detection, ratios of colposcopies performed per ≥CIN3 detection were calculated. For example, “14 hrHPV+ and DS+” describes a strategy whereby any case that is positive for any of the 14 hrHPV genotypes, and is also positive using DS testing, is sent for colposcopy.

This approach allowed an assessment of the trade‐offs between performing an additional triage test at the lab (e.g., cytology or DS testing) compared with performing additional colposcopies in the clinic (due to the estimates of referrals that would have occurred based on extended genotyping information).

## Results

Of the 7,727 evaluable primary screening women ≥25 years old in the ATHENA substudy with a valid CPR diagnosis, 314 had nonevaluable DS test results, 4,196 tested HPV negative and 878 had infections by more than one genotype. These were excluded from this analysis. A total of 2,339 women with valid results for Pap cytology, DS testing, HPV testing and genotype testing with single HPV genotype infections were available for analysis. A total of 171 CPR‐confirmed ≥CIN3 were identified (Fig. 1).

Table 1 presents the absolute risk of ≥CIN3 along with 95% CI attributable to each of the 14 hrHPV genotypes. HPV16 accounted for 83/171 (48.5%) of the ≥CIN3 cases, and a woman infected with HPV16 had a 19.3% probability of having ≥CIN3. HPV18 had about half this risk, but only accounted for ~10% of the cases. HPV31 and 33 each had a risk similar to HPV18, but HPV31 was 3.7 times more common in the ≥CIN3 patients than HPV33.

**Figure 2:**
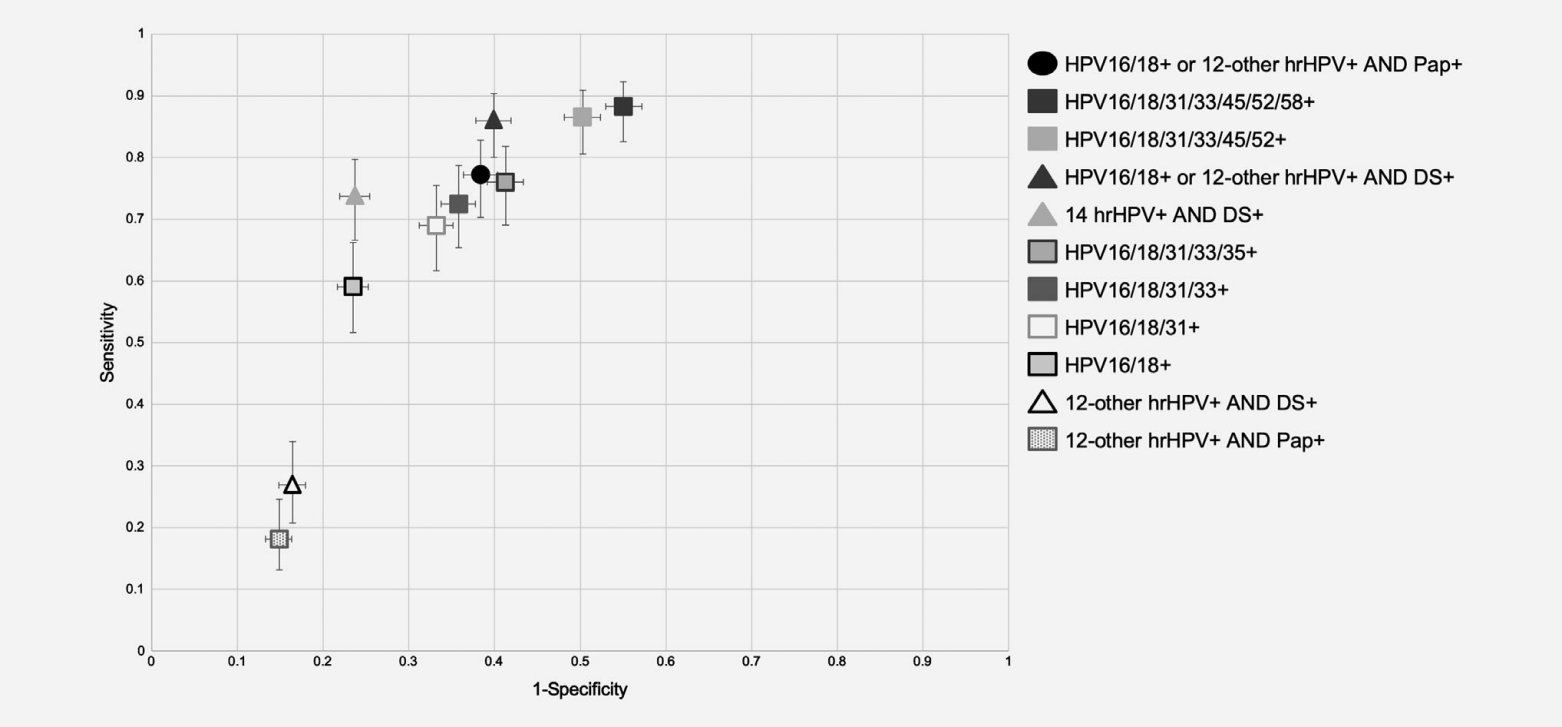
Performance of the 11 triage strategies in hrHPV‐positive women. The circle represents the FDA‐approved screening algorithm (HPV16/18+ or 12‐other hrHPV+ and Pap+) (Table 2). Triangles represent strategies that utilized DS testing. Squares represent strategies utilizing HPV genotyping only. Values are shown with 95% CIs. Abbreviations: DS, dual‐stain; HPV, human papillomavirus; hrHPV, high‐risk HPV.

The sensitivity and specificity estimates and performance ratios for the 10 triage strategies relative to the FDA‐approved algorithm are shown in Table 2, along with the difference, a measure of overall performance. Figure 2 shows these data plotted on a scatter chart with sensitivity on the y‐axis and 1‐specificity on the x‐axis. In addition, the sensitivity and specificity estimates and performance ratios of the 10 triage strategies for the detection of ≥CIN2 were calculated (Supplementary Table S1) and Supplementary Figure S1 shows these data plotted on a scatter chart with sensitivity on the y‐axis and 1‐specificity on the x‐axis. These results were comparable to those reported for detection of ≥CIN3.

**Table 2:** Comparison of different triage strategies of HPV‐positive women with the FDA‐approved primary screening algorithm for detection of ≥CIN3 using the ratio of sensitivities and 1‐specificities

Triage strategies	Sensitivity, %	Specificity, %	Ratio of sensitivities (95% CI)	Ratio of 1‐specificities (95% CI)	Difference (95% CI)
HPV16/18+ or 12‐other hrHPV+ AND Pap+ (FDA‐approved algorithm)	77.19	61.62			
HPV16/18/31/33/45/52/58+	88.30	44.97	1.14 (1.05–1.24)	1.43 (1.36–1.51)	0.29 (0.17–0.41)
HPV16/18/31/33/45/52+	86.55	49.72	1.12 (1.03–1.22)	1.31 (1.24–1.38)	0.19 (0.07–0.31)
HPV16/18+ or 12‐other hrHPV+ AND DS+	85.96	60.10	1.11 (1.04–1.19)	1.04 (0.99–1.09)	−0.07 (−0.16 to 0.01)
HPV16/18/31/33/35+	76.02	58.72	0.98 (0.91–1.06)	1.08 (1.02–1.13)	0.09 (−0.01 to 0.19)
14 hrHPV+ AND DS+	73.68	76.29	0.95 (0.86–1.05)	0.62 (0.57–0.67)	−0.34 (−0.45 to −0.23)
HPV16/18/31/33+	72.51	64.21	0.94 (0.86–1.02)	0.93 (0.88–0.98)	−0.01 (−0.10 to 0.09)
HPV16/18/31+	69.01	66.79	0.89 (0.82–0.97)	0.87 (0.82–0.91)	−0.03 (−0.12 to 0.06)
HPV16/18+	59.06	76.52	0.77 (0.69–0.84)	0.61 (0.58–0.64)	−0.15 (−0.23 to −0.07)
12‐other hrHPV+ AND DS+	26.90	83.58	0.35 (0.25–0.45)	0.43 (0.38–0.47)	0.08 (−0.03 to 0.19)
12‐other hrHPV+ AND Pap+	18.13	85.10	0.23 (0.16–0.31)	0.39 (0.36–0.42)	0.15 (0.07–0.23)

Abbreviations: CI, confidence interval; DS, dual‐stain; HPV, human papillomavirus; hrHPV, high‐risk HPV.

Table 3 shows baseline data from 11 different triage strategies utilizing the number of ≥CIN3 cases a given strategy detected from the 171 total cases as a benchmark. The efficiency of each strategy can be assessed by comparing the ratios of cases detected per number of colposcopies performed for each strategy. This table also contains data regarding DS testing as an alternative triage test alone or combined with partial HPV16/18 genotyping. The FDA‐approved algorithm, which utilizes Pap cytology for triage of the 12‐other hrHPV‐positive women, with direct referral of HPV16/18 positive women to colposcopy, detected 132/171 (77.2%) of the ≥CIN3. This algorithm is used as a reference for comparison. Detecting these 132 cases required 964 colposcopies, resulting in a rate of 7.3 colposcopies performed per ≥CIN3 detected. Using HPV genotypes 16/18 only for triage (as has been proposed in limited‐resource settings^41^), without referral of any of the 12‐other hrHPV positives to colposcopy, decreased ≥CIN3 detection to 101 cases (59.1%). However, the number of colposcopies also dropped, and the ratio was 6.0 colposcopies per ≥CIN3. Requiring positive cytology on 12‐other hrHPV‐positive women for triage only would have captured 31 of the 70 remaining cases at a ratio of 11.4 colposcopies per ≥CIN3 detected, a measure of the limited sensitivity and efficiency of cytology in this group of genotypes.

**Table 3:** Baseline disease detection using the 11 triage strategies in hrHPV‐positive women

Triage strategies	No. of ≥CIN3	No. of colposcopies	Colposcopies/CIN3
HPV16/18+ or 12‐other hrHPV+ AND Pap+ (FDA‐approved algorithm)	132	964	7.3
HPV16/18/31/33/45/52/58+	151	1,344	8.9
HPV16/18/31/33/45/52+	148	1,238	8.4
HPV16/18+ or 12‐other hrHPV+ AND DS+	147	1,012	6.9
HPV16/18/31/33/35+	130	1,025	7.9
14 hrHPV+ AND DS+	126	640	5.1
HPV16/18/31/33+	124	900	7.3
HPV16/18/31+	118	838	7.1
HPV16/18+	101	610	6.0
12‐other hrHPV+ AND DS+	46	402	8.7
12‐other hrHPV+ AND Pap+	31	354	11.4

The number of ≥CIN3 cases detected from the 171 total and the total number of colposcopies performed is shown for each triage strategy, along with the number of colposcopies required per ≥CIN3 case detected.

Abbreviations: ≥CIN3, cervical intraepithelial neoplasia ≥ grade 3; hrHPV, high‐risk human papillomavirus.

Performing DS testing on the 12‐other hrHPV‐positive women combined with HPV16/18 genotyping nearly maximizes cross‐sectional ≥CIN3 sensitivity at 86.0% (147/171). Although the colposcopy referral rate is increased, the ratio of colposcopies performed per ≥CIN3 detected is decreased to 6.9 (compared to 7.3) because of increased disease detection. The interplay between the importance of HPV16/18 genotyping and DS testing can be seen in the strategy of utilizing DS testing on all hrHPV‐positive women. There is a large increase in efficiency relative to the FDA‐approved algorithm, measured by both a reduction in the number of colposcopies to 640 as well as the ratio of colposcopies performed per ≥CIN3 detected to 5.1. This is accompanied by a nonsignificant loss in sensitivity.

The present study helps to inform the question of how many and which HPV genotypes would be required to refer women to colposcopy to at least meet the performance of the current FDA‐approved algorithm. Three combinations of genotypes standout: HPV16/18/31/33/35 would capture 130 cases, albeit with an increase in the number of colposcopies and a decrease in efficiency compared to the FDA‐approved algorithm as a reference. The combination of HPV16/18/31/33/45/52 rivals the sensitivity (148 *vs*. 147 cases) of the combined HPV16/18 and DS testing approach, albeit at a cost of 22% more colposcopies. Including seven of the hrHPV genotypes (HPV16/18/31/33/45/52/58) incorporated in the nonavalent HPV vaccine in a triage strategy detected three additional ≥CIN3 cases, but also increased the ratio for the number of colposcopies per ≥CIN3 detected from 8.4 to 8.9.

## Discussion

From a clinical perspective, finding and treating CIN3 is the key to cervical cancer prevention. How best to do this while minimizing clinical harm and minimizing the risk of untreated disease due to loss to follow‐up summarizes the current clinical “tension” regarding benefits *versus* harms in cervical cancer screening. Recognizing that perfection is not possible, all screening programs require periodic repeat examinations, and the time interval for repeat testing is determined by the long‐term risk of the occurrence of ≥CIN3 in women with a negative triage test result. However, strategies that maximize first pass ≥CIN3 detection, while minimizing colposcopic referral, are increasingly viewed as optimal in guideline discussions.

In the United States, the first FDA‐approved HPV primary screening algorithm was based on the cobas HPV Test that provides integrated HPV16/18 genotyping, and this algorithm has been incorporated into guideline discussions. The importance of HPV16 to algorithm performance cannot be overstated, as it accounts for ~50% of ≥CIN3 detected. In ATHENA, even a patient with negative for intraepithelial lesion or malignancy (NILM) cytology who is HPV16 positive has been found to be at substantial baseline risk for ≥CIN3 (~15%), well above the colposcopic referral threshold of ~5% at 3 years.^42^ While the risk associated with HPV18 is only about half that high at baseline, it remains substantial and most experts agree that this risk, combined with the strong association of HPV18 with cytologically hard‐to‐detect adenocarcinomas,^43^ makes triage to colposcopy for HPV18 important.

Given the above, adhering to the mantra of equal management for equal risk becomes increasingly important. The availability of reliable extended HPV genotyping tests has clarified the type‐specific risks associated with some genotypes whose prevalence/virulence interactions historically have been averaged or masked in the absence of genotypic information.^44^, ^45^, ^46^, ^47^, ^48^ In 2015, the cumulative incidence rates (CIRs) of ≥CIN3 during a 10‐year prospective cohort study of women aged 30+ in the Kaiser Permanente Northern California (KPNC) health system with NILM cytology at baseline for HPV16/18 and a pool of 11 other hrHPV genotypes were 20.7, 17.7 and 1.5, respectively.^49^ More recently, 3‐year CIRs were reported for a cohort of women at KPNC using more extensive genotyping.^45^ In that report, HPV16 was associated with a significantly higher risk than all other genotypes. However, the CIRs for HPV33, 31, 35 and 52 were comparable with HPV18, and significantly higher than HPV45, 58, 66, 51, 56, 39, 68 and 59.^45^ These data have helped to inform the design of more recent HPV screening and diagnostic tests.

However, extended genotyping clearly is not the only way to address the growing demand for an efficient and effective primary screening algorithm. The retrospective analysis from ATHENA demonstrated the potential for DS testing to provide remarkably superior triage compared with cytology, while still capitalizing on the power of HPV16/18 genotyping.^38^ Similarly, the recently published data from KPNC^20^ demonstrate that triage using DS testing provides improved risk stratification over 5 years compared with cytology, suggesting that in the KPNC setting DS testing may be a preferred triage test for HPV primary screening.

In the present analysis, the only strategy that resulted in greater sensitivity without a loss in specificity compared with the FDA‐approved algorithm was the “HPV16/18+ or 12‐other hrHPV+ and DS+.” The data shown in Table 3 suggest that this strategy could be an optimal compromise for clinical performance, and it is these types of data that have led to an ongoing U.S. FDA registration trial to clinically validate DS testing for clinical practice in the United States.

Although DS testing is less subjective than cytology, because the interpretation relies on identifying the presence or absence of an individual cell that has both a brown cytoplasm and a red nucleus rather than on morphologic interpretation, DS testing is not a molecular assay integrated into the HPV test, and requires going back to the original liquid‐based cytology vial collected from the HPV‐positive patients, preparing a slide and performing immunocytochemistry for professional interpretation. This is in part similar to Pap cytology in terms of specimen workflow, but no doubt will differ in reagent costs compared to a Pap stain.

With extended genotyping assays, the presence or absence of each genotype (or subsets of genotypes) is reported individually. Basing management on these alone can avoid the cost of an additional triage test but requires more colposcopies to find the same number of cases. Additionally, management by genotyping alone is based solely on risk without providing information regarding what is occurring within the cells. It has the potential to lead to delays in diagnosis for women who may have an infection with one of the 12‐other hrHPV genotypes associated with a lower risk but that is already on the verge of becoming a cancer. Various “costs” such as those of additional testing or risk in deferred identification (and the potential for loss to follow‐up) must be weighed against the algorithmic benefits of detecting more ≥CIN3, and the number of colposcopies needed per ≥CIN3 detected. This should lead to lively debate in future guideline development.

Finally, the data presented add to the evolving awareness of how HPV vaccination can directly impact screening. Most of the screening algorithms today are driven by disease caused by HPV16 and HPV18. As these viruses are removed from the population, the risk associated with a positive screening test decreases, as most of the overall risk is prevalence driven. Thus, strategies incorporating DS testing, which provide genotype agnostic risk information, or strategies that provide specific individual genotypes, may potentially both become more useful, or a test such as DS that “assembles” risk of neoplasia may become preferable.

### Strengths and limitations

A major strength of this substudy is that it is derived from ATHENA, a large clinical validation study with thorough and rigorous disease assessment with adjudicated disease endpoints. One limitation is that the present analysis was restricted to single‐genotype infections; however, sensitivity of the FDA‐approved algorithm to detect ≥CIN3 in this ATHENA study subpopulation was 75.3%, compared to 74.5% in the total ATHENA population.^36^ Furthermore, this analysis utilized results from LINEAR ARRAY genotyping, which, while similar to the cobas HPV Test in many performance characteristics,^46^ and clinically validated according to the Validation of HPV Genotyping Tests framework,^50^ is a different test than the FDA‐approved cobas HPV Test. In addition, liquid‐based cytology vials used for CINtec PLUS Cytology had been stored for up to 5 years before testing. These liquid‐based cytology specimens were from the second of two consecutive samples collected at the initial screening visit. Thus, the values described here for DS testing may underestimate the performance of the test relative to data derived from ongoing clinical trials.

In summary, as the world moves toward HPV primary screening, optimal triage is needed to maximize precancer detection while minimizing colposcopy. The data presented in this retrospective analysis demonstrate how genotyping and DS testing can be used to approach this goal of optimization in a data‐driven manner.

## Supporting information


**Appendix S1**: Supplementary Material

## Data Availability

The data that supports the findings of our study are available from the corresponding author upon reasonable request.
