# Supplementary material for: Approaches to triage optimization in HPV primary screening: Extended genotyping and p16/Ki‐67 dual‐stained cytology—Retrospective insights from ATHENA
Source: Int J Cancer. 2019 Oct 6;146(9):2599–607. doi: 10.1002/ijc.32669 (PMC7078939; doi:10.1002/ijc.32669)
Supplement: Supplementary file 1 — Appendix S1: Supplementary Material [file IJC-146-2599-s001.docx]

**ONLINE SUPPLEMENT**

**Approaches to triage optimization in HPV primary screening: Extended genotyping and p16/Ki-67 dual-stained cytology − retrospective insights from ATHENA**

# TITLE PAGE

**Authors**: Mark H. Stoler^a^, Ed Baker^b^, Sean Boyle^b^, Shagufta Aslam^b^, Ruediger Ridder^c^, Warner K. Huh^d^, Thomas C. Wright Jr.^e^

**Affiliations**: ^a^Department of Pathology, University of Virginia Health System, Charlottesville, VA, USA; ^b^Roche Molecular Systems Inc., Pleasanton, CA, USA; ^c^Ventana Medical Systems, Inc. (Roche Tissue Diagnostics), Tucson, AZ, USA; ^d^Division of Gynecologic Oncology, University of Alabama at Birmingham, Birmingham, AL, USA; ^e^Department of Pathology, Columbia University, New York City, NY, USA

**Short title**: Extended genotyping versus dual-stain cytology for triage

**Corresponding author**:

Mark H. Stoler, MD

Professor Emeritus of Pathology and Clinical Gynecology

Department of Pathology

University of Virginia Health System

1215 Lee Street

Charlottesville, VA 22908, USA

Phone: +1 (434) 982 0284; Fax: +1 (434) 924 9492; E-mail: mhs2e@virginia.edu

# SUPPLEMENTARY TABLES

## Table S1. Comparison of different triage strategies of HPV-positive women with the FDA-approved primary screening algorithm

## for detection of ≥CIN2 using the ratio of sensitivities and 1-specificities

| **Triage strategies** | **Sensitivity, %** | **Specificity, %** | **Ratio of sensitivities  (95% CI)** | **Ratio of 1-specificities  (95% CI)** | **Difference (95% CI)** |
| --- | --- | --- | --- | --- | --- |
| HPV16/18+ or 12-other hrHPV+ AND Pap+ (FDA-approved algorithm) | 71.98 | 62.58 |  |  |  |
| HPV16/18/31/33/45/52/58+ | 84.05 | 45.82 | 1.17 (1.07–1.27) | 1.45 (1.37–1.53) | 0.28 (0.15–0.41) |
| HPV16/18+ or 12-other hrHPV+ AND DS+ | 81.32 | 61.43 | 1.13 (1.06–1.20) | 1.03 (0.98–1.08) | -0.10 (-0.19–-0.01) |
| HPV16/18/31/33/45/52+ | 79.77 | 50.38 | 1.11 (1.01–1.20) | 1.33 (1.25–1.40) | 0.22 (0.10–0.34) |
| HPV16/18/31/33/35+ | 72.76 | 59.75 | 1.01 (0.93–1.09) | 1.08 (1.02–1.14) | 0.06 (-0.03–0.16) |
| 14 hrHPV+ AND DS+ | 68.09 | 77.67 | 0.95 (0.85–1.04) | 0.60 (0.55–0.65) | -0.35 (-0.45–-0.24) |
| HPV16/18/31/33+ | 66.93 | 65.03 | 0.93 (0.85–1.01) | 0.93 (0.88–0.99) | 0.00 (-0.09–0.10) |
| HPV16/18/31+ | 64.59 | 67.72 | 0.90 (0.82–0.98) | 0.86 (0.81–0.91) | -0.03 (-0.13–0.06) |
| HPV16/18+ | 50.19 | 76.90 | 0.70 (0.63–0.76) | 0.62 (0.58–0.65) | -0.08 (-0.15–-0.01) |
| 12-other hrHPV+ AND DS+ | 31.13 | 84.53 | 0.43 (0.34–0.52) | 0.41 (0.37–0.46) | -0.02 (-0.12–0.08) |
| 12-other hrHPV+ AND Pap+ | 21.79 | 85.69 | 0.30 (0.24–0.37) | 0.38 (0.35–0.42) | 0.08 ( 0.01–0.15) |

CI, confidence interval; DS, dual-stain; HPV, human papillomavirus; hrHPV, high-risk HPV.

# SUPPLEMENTARY FIGURES

## Figure S1. Performance of the 11 triage strategies for detection of ≥CIN2 in hrHPV-positive women

DS, dual-stain; HPV, human papillomavirus; hrHPV, high-risk HPV.

The circle represents the FDA-approved screening strategy (HPV16/18+ or 12-other hrHPV+ and Pap+) (Table 2). Triangles represent strategies that utilized DS testing. Squares represent strategies utilizing HPV genotyping only. Values are shown with 95% CIs.
